# Degrading products of chondroitin sulfate can induce hypertrophy-like changes and MMP-13/ADAMTS5 production in chondrocytes

**DOI:** 10.1038/s41598-019-52358-4

**Published:** 2019-11-01

**Authors:** Youn-Kwan Jung, Hye-Ri Park, Hyun-Jung Cho, Ji-Ae Jang, Eun-Ju Lee, Min-Su Han, Gun-Woo Kim, Seungwoo Han

**Affiliations:** 1 Biomedical Research Institute, Gyeongsang National University Hospital, Jinju, Gyeongsangnam-do Republic of Korea; 2 Laboratory for arthritis and bone biology, Fatima Research Institute, Daegu Fatima hospital, Daegu, Republic of Korea; 3 Department of Internal medicine, Daegu Fatima Hospital, Daegu, Republic of Korea; 4 Department of Internal medicine, Kyungpook National University Hospital, Daegu, Republic of Korea

**Keywords:** Mechanisms of disease, Cartilage

## Abstract

Chondroitin sulfate (CS) is the most abundant glycosaminoglycan (GAG) in articular cartilage and the loss of CS-GAG occurs early in OA. As a major component of perichondral matrix interacting directly with chondrocytes, the active turnover of CS can affect to break the homeostasis of chondrocytes. Here we employ CS-based 3-dimensional (3D) hydrogel scaffold system to investigate how the degradation products of CS affect the catabolic phenotype of chondrocytes. The breakdown of CS-based ECM by the chondroitinase ABC (ChABC) resulted in a hypertrophy-like morphologic change in chondrocytes, which was accompanied by catabolic phenotypes, including increased MMP-13 and ADAMTS5 expression, nitric oxide (NO) production and oxidative stress. The inhibition of Toll-like receptor 2 (TLR2) or TLR4 with OxPAPC (TLR2 and TLR4 dual inhibitor) and LPS-RS (TLR4-MD2 inhibitor) ameliorated these catabolic phenotypes of chondrocytes by CS-ECM degradation, suggesting a role of CS breakdown products as damage-associated molecular patterns (DAMPs). As downstream signals of TLRs, MAP kinases, NF-kB, NO and STAT3-related signals were responsible for the catabolic phenotypes of chondrocytes associated with ECM degradation. NO in turn reinforced the activation of MAP kinases as well as NFkB signaling pathway. Thus, these results propose that the breakdown product of CS-GAG can recapitulate the catabolic phenotypes of OA.

## Introduction

Osteoarthritis (OA) is the most common degenerative disease of joint characterized by hypertrophic differentiation of chondrocytes that leads to apoptosis in response to excessive mechanical stress^1^. Hypertrophic chondrocytes also produce the proteases such as MMP-13 and ADAMTS5 which are responsible for the cartilage extracellular matrix (ECM) degradation that is a central feature of OA^2^. However, this hypertrophy-centered dogma in pathogenesis of OA is being challenged. Evidences showed that the treatment of bioactive type II collagen (Col2) fragment itself leads to hypertrophic change and apoptosis of chondrocytes in articular cartilage organ culture^3^, suggesting a mechanical stress- or enzyme-mediated ECM degradation might regulate the overall phenotypes of OA including chondrocyte hypertrophy^4,5^.

The matrix of hyaline articular cartilage is comprised primarily of collagen, proteoglycans and water molecules^6,7^. Col2 which is the most abundant collagen protein in cartilage ECM has a role of framework providing tensile strength to articular cartilage. The most abundant proteoglycan is aggrecan which is composed of chondroitin sulfate and keratan sulfate binding to the linear core protein and is connected to hyaluronic acid backbone^6^. In the progression of OA, the major proteinases involved in cartilage ECM destruction are MMP-13 for collagen and ADAMTS4 and 5 for aggrecan^8^. Active remodeling of cartilage ECM by these proteases can induce a variety of breakdown product of ECM such as fragmented proteins and glycosaminoglycans (GAGs) which can function as damage-associated molecular patterns (DAMPs)^9,10^. Such molecules known to act as extracellular DAMPs include biglycan, fibronectin, low-molecular weight hyaluronic acid and tenascin C in OA^11–13^. DAMPs activate pattern recognition receptors including Toll-like receptors (TLRs) and inflammasomes which are likely to be involved in the progression of OA through inducing low-grade inflammation and production of proteases^14,15^.

Chondroitin sulfate (CS) makes up the main constituent of cartilage proteoglycan as a member of sulfated GAGs that are long unbranched polysaccharides. Spectroscopy analysis revealed that CS content accounts for 35% weight/dry weight (w/dw) of young articular cartilage and 20% w/dw of adult articular cartilage of human^16^. In addition to its composition in cartilage, CS has a profound interaction capacity with individual chondrocyte. Chondrocyte-associated matrix GAG analysis revealed that CS has 5- to 7-times more chondrocyte binding compared to hyaluronic acid and keratin sulfate^17^. The ECM just surrounding the chondrocytes, termed perichondral matrix, has a higher proteoglycan turnover rate compared to interterritorial ECM^18^. Actually, the changes in collagen and proteoglycan distribution of perichondral matrix are observed in the early stage of OA which precede chondrocyte proliferation and cell cluster formation^19^. As a major component of perichondral matrix, the active turnover of CS may not only induce changes of the mechanical property of perichondral matrix but may also act as critical stimuli to break the homeostasis of chondrocytes^20^. In the present study, we investigated the effects of CS breakdown on chondrocytes and molecular mechanisms involved in the catabolic phenotypes using CS-based 3-dimensional (3D) hydrogels embedded with chondrocytes.

## Results

### CS-based ECM degradation induces a hypertrophy-like morphological change in chondrocytes which is accompanied by an enhanced MMP-13 and ADAMTS5 expression and oxidative stress

First, we checked the phenotypic characteristics of primary chondrocytes under the CS-based 3D hydrogel scaffolds culture condition. GAG contents stained with Safranin-O was increased by 3rd week and then decreased from 5th week. This decrease of GAG was accompanied by the hypertrophy-like morphologic change of chondrocytes in H&E staining. Chondrogenic genes such as Sox9, Col2, and aggrecan (Agc) was decreased until 5th week, but increased to 7th week. Ihh, a marker for pre-hypertrophic chondrocytes, was increased at 3rd week. Hypertrophic marker such as type X collagen (Col10), MMP-13 and Runx2, and osteoblast marker, type I collagen a1 (Col1a1) was increased at 7th week (Supplementary Fig. 1). These results suggest that our 3D hydrogel culture condition is suitable to induce chondrocyte maturation, and at least 3 week of culture does not induce hypertrophic differentiation of chondrocytes.

**Figure 1:**
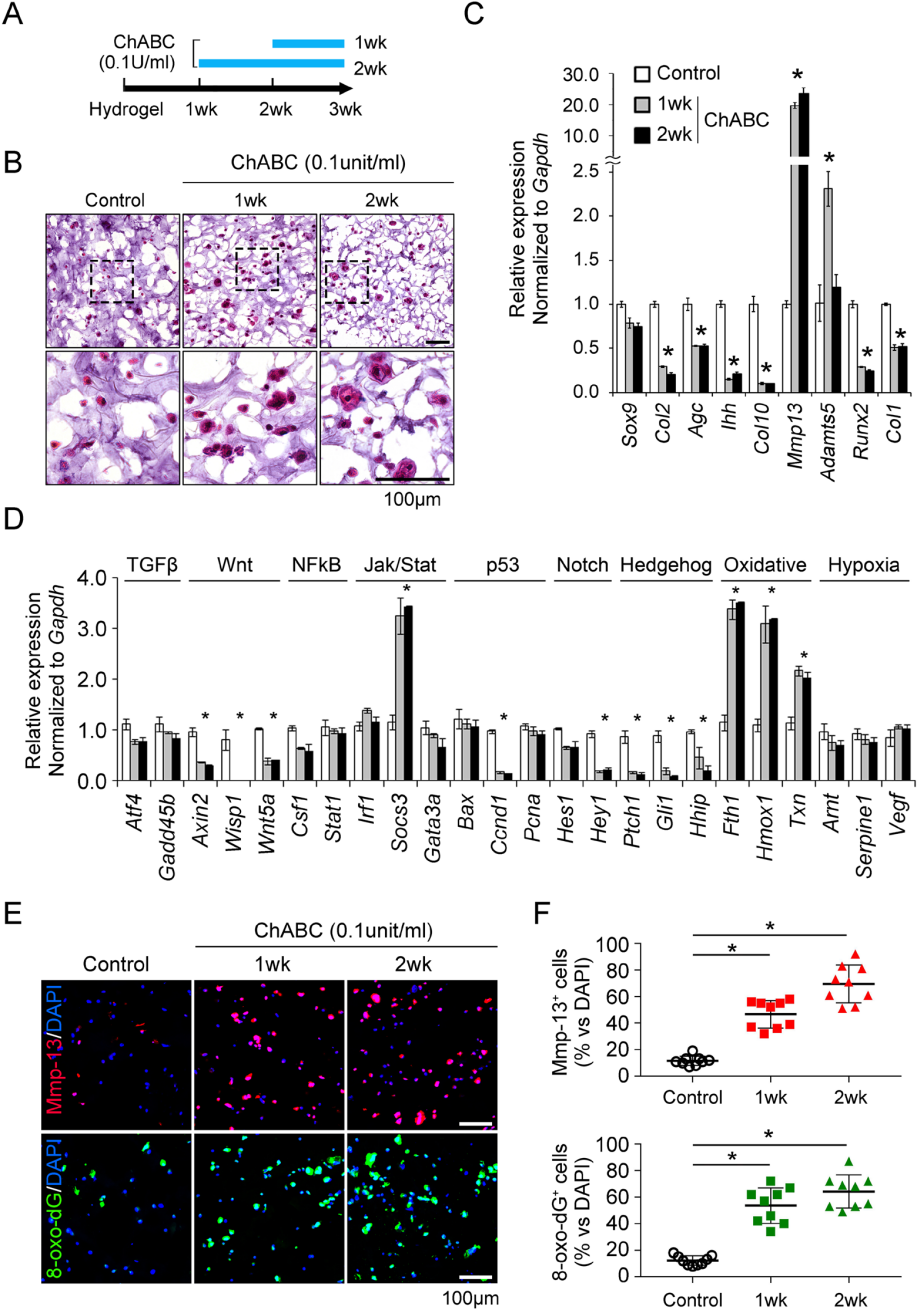
Cleavage of the extracellular matrix (ECM) alone can lead to the hypertrophy-like changes and an increase of MMP-13, ADAMTS5 and oxidative stress in primary chondrocytes. (**A**) The time schedule of the chondroitinase treatment in chondroitin sulfate (CS)-based 3 dimensional (**D**)-hydrogel culture system. Primary chondrocytes obtained from E15.5 long bone were cultured at a density of 1.5 × 10^7^ cells/mL in 3D-hydrogel composed of 3% (w/v) CS and 5% (w/v) polyethylene glycol (PEG) for 3 weeks. Chondroitinase was treated during one or two week as indicated. (**B**) Hematoxylin-eosin (H&E) staining for overall chondrocyte morphology within the 3D-hydrogel. The area within the black rectangle in the upper column was enlarged in the lower column. Scale bar indicates 100 μm. (**C**) Quantitative RT-PCR of chondrocyte-specific marker genes for the chondrogenic differentiation such as Sox9, Col2 and Agc, pre-hypertrophy of Ihh, early hypertrophy of Col10 and Runx2, late hypertrophy of MMP-13 and ADAMTS5, and osteoblast of Col1a1. (**D**) RNA expression of the target genes involved in chondrocyte-associated major signaling pathways. *P < 0.05 vs. the control group. (**E**) The representative images of MMP-13 and 8-oxo-dG immunofluorescence staining for chondrocyte hypertrophy and oxidative stress, respectively. (**F**) Quantification of MMP-13 or 8-oxo-dG-positive cells which was expressed as a percentage of positively stained cells among total cells. *P < 0.05 vs. the control group.

To elucidate the role of ECM degradation on chondrocytes, chondrotinase ABC (ChABC) was treated in CS-based 3D hydrogel culture system for 1 wk and 2 wk as indicated in Fig. 1A. Interestingly, the breakdown of chondroitin sulfate ECM has made the matrix-embedded chondrocytes hypertrophic which look like the volume of cytoplasm has increased (Fig. 1B). When we assessed the expression of chondrocyte marker genes, MMP-13 was notably increased by ChABC and ADAMTS5 was increased by 1 wk treatment and decreased by 2 wk of ChABC. On the other hand, the chondrogenic markers such as Col2 and Agc, the prehypertrophic marker, Ihh, the early hypertrophic markers, Col10 and Runx2, and the osteoblast marker, Col1 was decreased by ECM breakdown in RNA level (Fig. 1C).

To evaluate the effect of signaling pathways according to the breakdown of ECM, we assessed the expression of target molecules regulated by major signaling pathways of chondrocyte biology. *Socs3* RNA expression increased significantly meaning the activation of STAT3 signaling, and the expression of oxidative stress markers such as Fth1, Hmox1, and Txn all increased. However, the target molecules of Wnt, Notch, Hedgehog signaling rather decreased, and the cellular proliferation marker, cyclinD1 was also reduced. There was no difference in the gene expression associated with TGFβ, NFkB, and hypoxia (Fig. 1D). Immunofluorescence staining to quantify the relative level of a protein revealed that the ECM degradation by ChABC enhanced the expression of MMP-13 as well as 8-oxo-dG, the marker for oxidative stress (Fig. 1E,F). Based on the increase of genes involving oxidative stress, we hypothesized that the alterations in chondrocyte metabolism associated with ECM degradation can affect the production of oxidative stress and proteases production. The expression of the endogenous antioxidant *Sod2* has increased, but there has been no change in the expression of genes involved in oxidative phosphorylation and glycolysis, suggesting that the ECM degradation does not significantly affect the chondrocyte metabolism (Supplementary Fig. 1).

### The breakdown product of CS generated by chondroitinase act as damage associated molecular patterns (DAMPs) through TLR2 and TLR4

**Figure 2:**
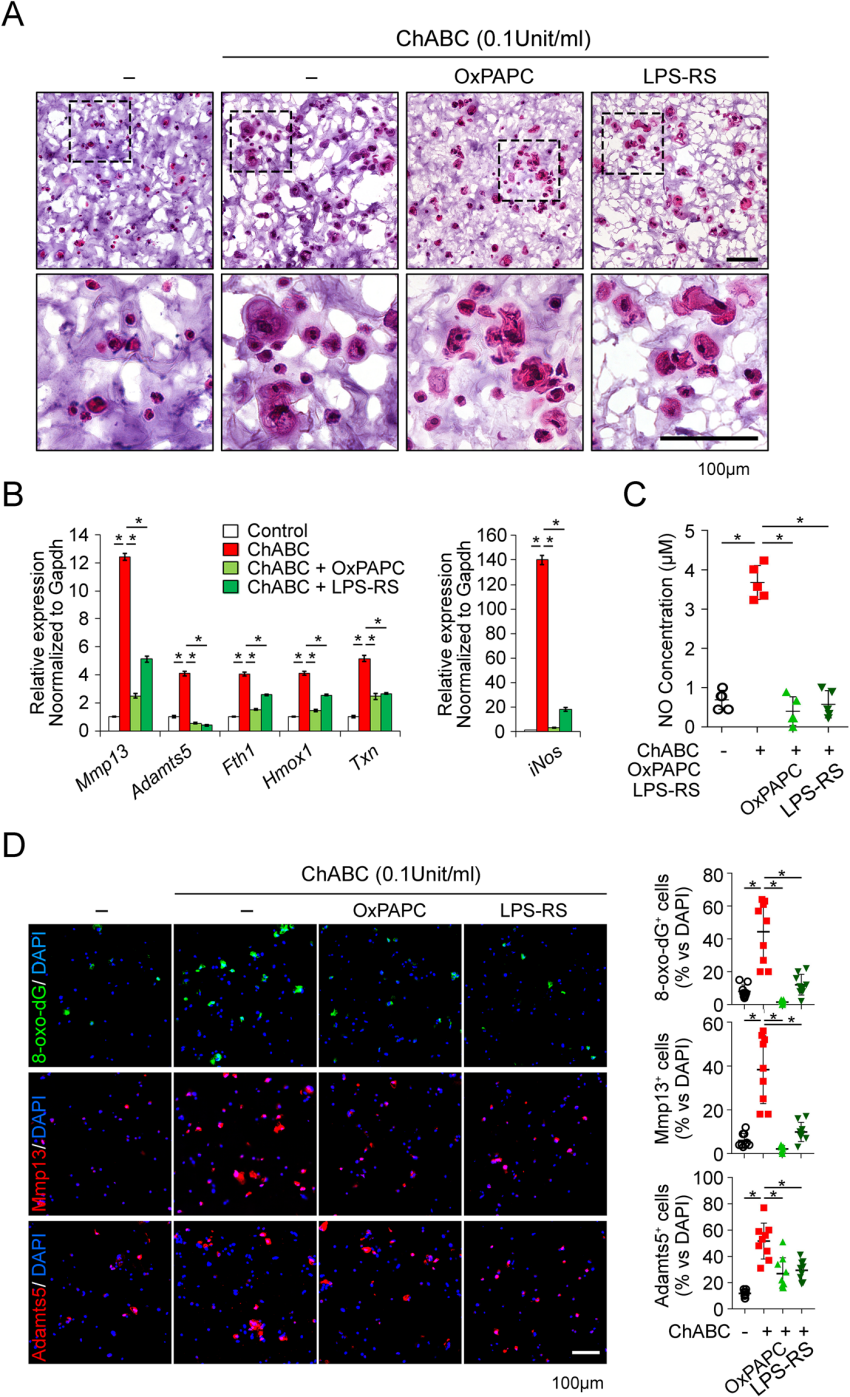
Breakdown of chondroitin-sulfate-based ECM increases the production of MMP-13, ADAMTS5, oxidative stress and nitric oxide (NO) through TLR2 and TLR4 in 3D-cultured chondrocytes. (**A**) H&E staining for gross morphology of chondrocytes. Primary chondrocytes cultured in chondroitin-sulfate-based 3D-hydrogel for 2 wks were treated with chondroitinase ABC in the presence of OxPAPC (TLR2 and TLR4 dual inhibitor) or LPS-RS (TLR4-MD2 inhibitor) for 1 wk. Black rectangles in each panel denote areas enlarged in insets. Scale bar represents 100 μm. (**B**) Real time qRT-PCR for the RNA expression of indicated target genes. MMP-13 and ADAMTS5 are the major matrix degrading protease and Fth1, Hmox1 and Txn are the markers for oxidative stress. *P < 0.05. (**C**) The supernatant of 3D-hydrogel culture collected from different groups were analyzed for NO concentration measured by measuring the absorbance at the 520 nm wavelength. *P < 0.05. (**D**) The representative images of immunofluorescence staining for 8-oxo-dG, MMP-13 and ADAMTS5 in frozen-sectioned 3D-hydrogel. The percentage of cells positive for target staining is indicated as dot graph in right column. *P < 0.05 and **P < 0.01.

Next, we tested if the breakdown products of chondroitin-sulfate can function as damage-associated molecular patterns (DAMPs) that can be responsible for the increase of proteases and oxidative stress. In the preceding study, we confirmed that the ligands for TLR2 and TLR4 significantly involves in the expression of MMP-3 and MMP-13 in chondrocytes^21^. On this basis, we inhibited the function of TLR2 and TLR4 with OxPAPC, a dual TLR2 and TLR4 inhibitor or LPS-RS, a functional TLR4 inhibitor through MD2 inhibition, and assessed the chondrocyte phenotype by chondroitinase treatment. Morphologically, OxPAPC and LPS-RS partially inhibited the hypertrophic change of chondrocytes by chondroitinase-induced ECM degradation (Fig. 2A). The increase in MMP-13 and ADAMTS5 due to the breakdown of ECM was significantly reduced by both OxPAPC and LPS-RS. Not only that, OxPAPC and LPS-RS significantly suppressed the increase of oxidative stress markers such as Fth1, Hmox1, and Txn (Fig. 2B). Inducible nitric oxide synthase (iNOS) well known to be critical for cartilage degeneration was significantly increased in RNA level by ECM degradation by chondroitinase, which was nearly completely suppressed by TLR2 and TLR4 inhibition by OxPAPC and LPS-RS (Fig. 2B). The NO levels in culture supernatant significantly reduced by TLR2 and TLR4 antagonists as well (Fig. 2C). Immunofluorescence staining revealed that the 8-oxo-dG, MMP-13 and ADAMTS5 that were significantly induced by ECM breakdown were considerably suppressed by OxPAPC and LPS-RS (Fig. 2D). These results indicate that the breakdown products of CS function as DAMPs through TLR2 and TLR4 which is responsible for the increase of proteases such as MMP-13 and ADMATS5, and oxidative stress.

### Effects of ECM degradation on chondrocyte phenotypes depends on MAP kinases, NFkB, STAT3 and NO signaling

**Figure 3:**
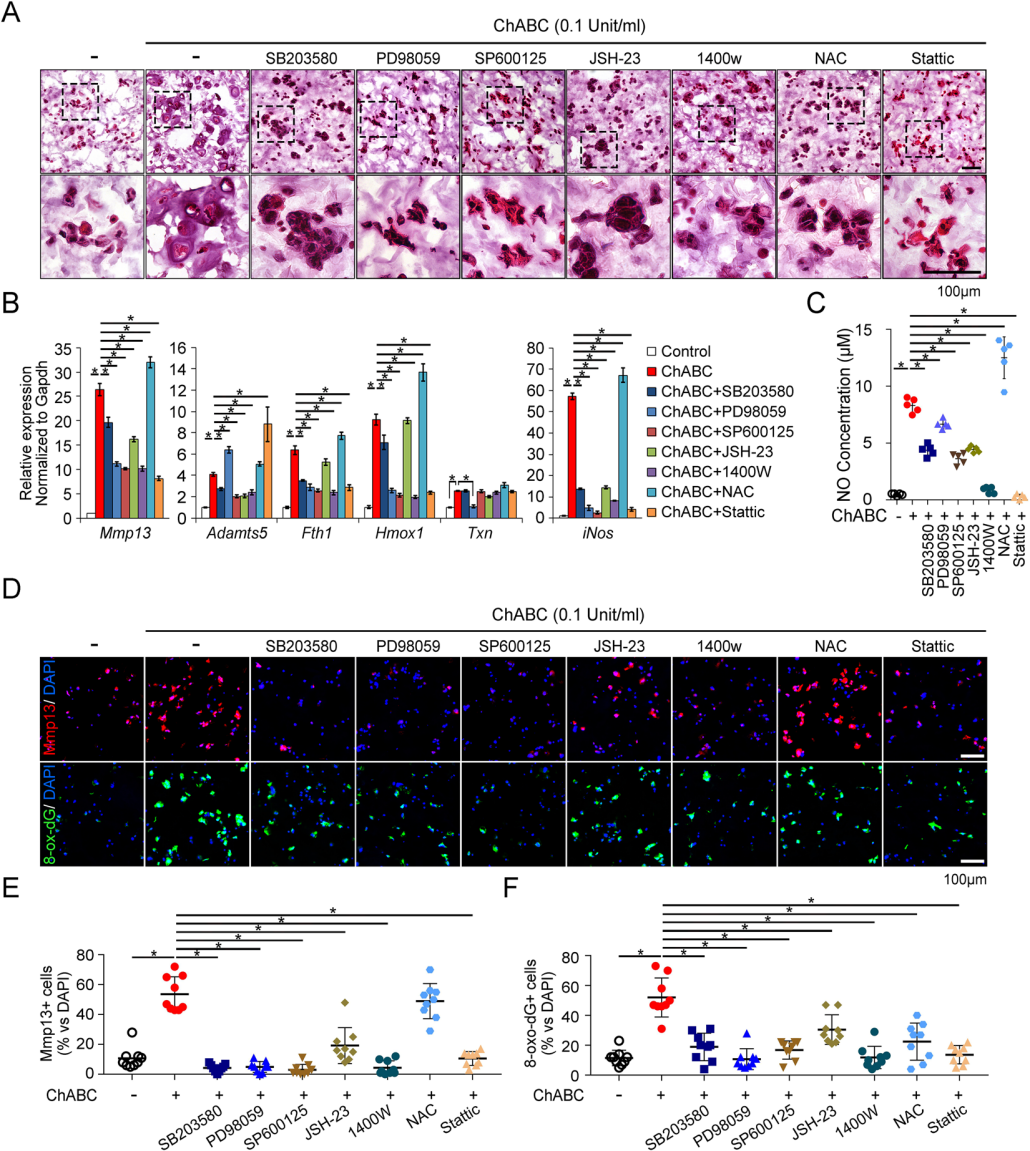
Catabolic changes of chondrocyte mediated by ECM degradation are dependent on the activation of MAP kinases, NF-kB, STAT3 and NO signaling. (**A**) Representative images of H&E staining of frozen-sectioned 3D-hydrogels which were treated with chondroitinase ABC in the presence of various signaling inhibitors for p38 (SB203580), ERK1/2 (PD98059), JNK MAPKs (SP600125), NFkB (JSH-23), iNOS (1400 w), oxidative stress (NAC), and STAT3 (Stattic). (**B**) Real-time qRT-PCR analysis of indicated genes in 3D-hydrogel embedded chondrocytes treated with chondroitinase ABC and various signaling inhibitors. *P < 0.05. (**C**) NO concentration in the supernatant of 3D hydrogel culture was determined by measuring the absorbance at the 520 nm wavelength. *P < 0.05. (**D**) Representative images of immunofluorescence staining for MMP-13 (Red) and 8-oxo-dG (Green). DAPI was used to stain the nuclei (blue). The scale bar represents 100 μm. (**E**,**F**) MMP-13 or 8-oxo-dG-positive cells were quantified as the percentage of target-positive cells/DAPI positive total cells, in 9 random microscopic high-power fields (×200 objective).

We then conducted inhibitor assays to identify which TLR downstream signaling is involved in the CS-ECM degradation-related chondrocyte phenotype. The MAP kinases and NFkB signaling that is the major downstream signaling of TLR2 and TLR4 was inhibited with SB203580 for p38 MAPK, PD98059 for MEK1/ERK, SP600125 for JNK and JSH-23 for NFkB. NO production by iNOS was inhibited with 1400 W, oxidative stress with N-acetyl-cysteine (NAC) and STAT3 activity with Stattic, which was applied based on the increase of Socs3 by ChABC (Fig. 1C). These inhibitors except JSH-23 and NAC significantly inhibited the hypertrophy-like morphologic changes of chondrocytes by ECM degradation (Fig. 3A). In addition, ChABC-mediated MMP-13 RNA expression was suppressed by these inhibitors except antioxidant NAC which rather increased MMP-13 expression. The ADAMTS5 expression was decreased by inhibitors of p38 and JNK MAP kinases, NFkB and NO inhibition in RNA level, while it was increased by ERK1/2 and STAT3 inhibitors. The expression of Fth1 and Hmox1, the oxidative stress markers was decreased by inhibitors, but was not affected by JSH-23 and rather increased by NAC. The other oxidative stress marker Txn was reduced only by ERK1/2 MAP kinase inhibitor. The iNOS expression was reduced by all inhibitors except NAC which increased iNOS significantly (Fig. 3B). This result has been reproduced in the NO concentration assay of 3D-hydrogel culture supernatant (Fig. 3C). The expression of MMP-13 assessed by the immunofluorescence staining was significantly attenuated by the inhibitors for p38, ERK1/2, JNK MAP kinase, NFkB, iNOS and STAT3 while it was not affected by the antioxidant, NAC. All these inhibitors suppressed the expression of oxidative stress marker, 8-oxo-dG (Fig. 3D). These results suggest the MAP kinases, NFkB, NO and STAT3-related signaling play an important role in the catabolic phenotype of chondrocytes associated with ECM degradation.

### NO reinforces the activation of MAP kinases as well as NFkB signaling

**Figure 4:**
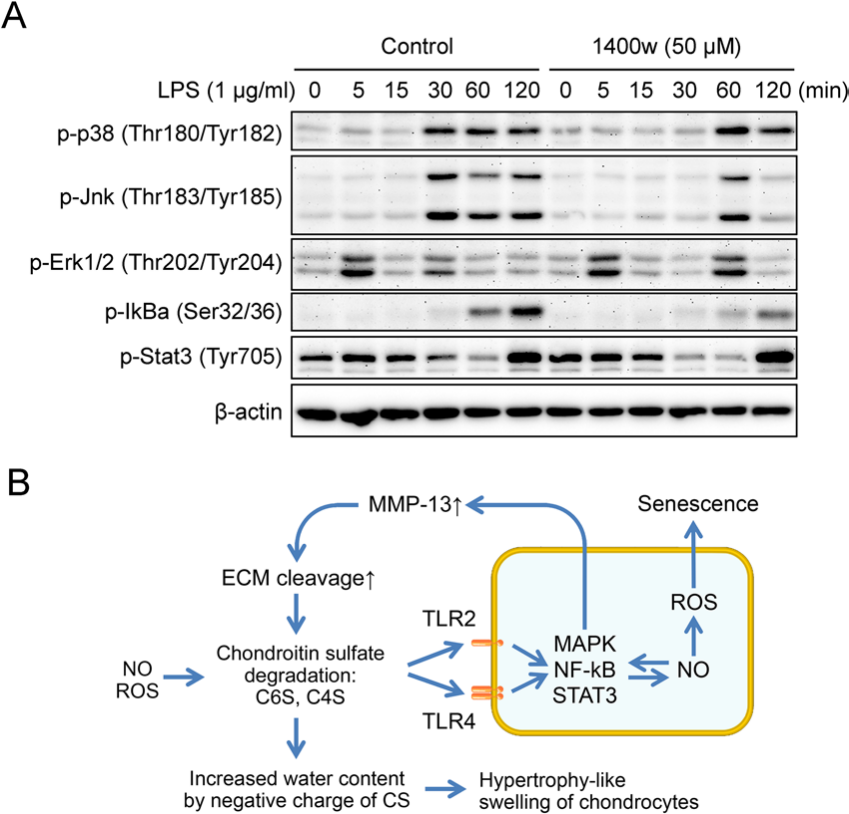
Inhibition of NO attenuates the phosphorylation of MAP kinases and NFkB signaling, but not STAT3 signaling. (**A**) Time-course phosphorylation of MAP kinases, IkBa and STAT3 by LPS in the presence or absence of 1400 w, an iNOS inhibitor. Primary chondrocytes were starved overnight and pretreated with 1400 w for 15 min followed by stimulation with 1 μg/ml of LPS for indicated time. (**B**) Schematic diagram depicting the catabolic mechanism of chondrocytes by the breakdown of chondroitin-sulfate-based ECM. The degradation of chondroitin-sulfate can produce a new ligand for TLR2 and TLR4 such as C6S and C4S which can function as DAMPs. The activated TLRs can induce the expression of MMP-13 and ADAMTS5 through MAP kinases, NFkB and STAT3 signaling forming a positive feedback loop with iNOS-NO system. This increased MMP-13 and ADAMTS5 can amplify the degradation of ECM causing a vicious cycle of cartilage destruction. In addition, the exposure of sulfate residue in CS with negative charge can induce an increase of water content resulting in a hypertrophy-like swelling of chondrocytes.

To elucidate the interaction between NO and other catabolic signaling pathways such as MAP kinases, NFkB and STAT3 in TLR2 and TLR4 activation, we assessed the effect of iNOS inhibition with 1400 w on the LPS-mediated phosphorylation of each signaling in monolayer culture condition of primary chondrocytes. The LPS treatment in primary chondrocytes phosphorylated the p38 and JNK MAP kinases in about 30 min, but the inhibition of iNOS-NO system delayed its activation. In addition, NO inhibition had no effect on the first activation of ERK1/2 MAP kinase seen in 5 min, but also delayed its second activation in 30 min. The phosphorylation of IkB, the component of canonical NFkB signaling, was significantly suppressed by NO inhibition. However, STAT3 phosphorylation was not affected by NO inhibition (Fig. 4A). These results suggest that MAP kinases and NFkB signaling not only increase iNOS-mediated NO production, but also have a positive feedback loop with NO signaling where NO enhances the activation of MAP kinases and NFkB signaling.

## Discussion

The most interesting finding of our study is that the breakdown of CS-GAG results in the hypertrophy-like morphologic change of chondrocytes without the increase of hypertrophy-related genes such as Runx2, Ihh and Col10 (Fig. 1B,C). The current mainstream opinion regarding the pathogenesis of OA is centered on the chondrocyte hypertrophy that is preceded to chondrocyte apoptosis and is needed to increase of proteases such as MMP-13 and ADAMTS5^2^. Despite of the morphological similarity, our data suggest a difference between the hypertrophy-like change associated with ECM degradation and hypertrophic differentiation during endochondral bone formation. Recent study identified two different mechanism of chondrocyte volume enlargement in growth plate; the first is true cellular hypertrophy that maintains its initial high density of dry mass by a proportional increase of dry mass product and fluid intake. This is followed by second phase of swelling of chondrocyte by disproportionate fluid uptake, which is critical for regulating growth rate of long bone^22^. Actually, 3D hydrogel specimens treated with ChABC revealed more space in ECM, suggesting an increase of water content and it was associated with the volume of individual chondrocytes.

The fixed negative charges on the GAG chain are critical for maintaining the water content in tissue^23^. The water content of OA cartilage is increased despite the decreased chondroitin sulfate concentration^24^. This can be explained by the enhanced reactivity with sulfate- and carboxyl-group of the CS in OA cartilage, indicating increased exposure of negative charge in CS-GAG chain by mechanical stress- or enzyme-mediated degradation^24–26^. This increase of water content associated with GAG degradation can induce a swelling of individual chondrocytes in OA cartilage making chondrocytes vulnerable to mechanical stress^27^. Our results propose indirect evidence that the breakdown of CS-GAG and as a result increase of water content in cartilage ECM can induce a hypertrophy-like morphologic change and subsequent vulnerability of chondrocytes in OA.

A further interesting finding of this study is that the degrading product of CS-GAG acts as a DMAPs being responsible for the increase of MMP-13 and ADAMTS5 through TLR2 and TLR4. There is growing evidences that a large number of cellular proteins can be proteolytically released in the process of OA upon mechanical stress and they can trigger and accentuate cartilage damage as DAMPs^9,28^. These are largely divided into two categories of intracellular DAMPs including high-mobility group protein B1 (HMGB1), and alarmins (S100A8, and S100A9), and extracellular DAMPs, heparan sulfate and low molecular weighted hyaluronan^9,28,29^. Our data showed the ChABC-mediated breakdown product of CS act as DAMPs through TLR2 and TLR4 (Fig. 2). CS is the most abundant GAG in cartilage as a component of aggrecan, biglycan, decorin, and fibromodulin^30^. CS mainly consists of two repeating disaccharide units, chondroitin-6-sulfate (C6S) and C4S according to the position of sulfate residues at 4- or 6-O-positions of N-acetylgalactosamine. The CS degrading product of C6S and C4S in synovial fluid was reported to have increased in the early stage of OA, which gradually decreased as OA progresses^31^. In addition, cartilage specimen of OA patients revealed a significant reduction in CS content and chain length in proportion to the severity of OA^24,32^. Together with these evidences, our data suggest that the breakdown products of CS-GAG actively generated in early stage of OA can function as DAMPs through TLR2 and TLR4 and play an important role in the progress of cartilage degeneration.

However, it is poorly understood what kind of endogenous enzyme works in CS degrading process. As human has no chondroitinase, the hyaluronidases (HYALs) are considered to be the enzymes acting at the initial stage of the degradation process because hyaluronic acid (HA) is similar in structure to non-sulfated CS. Among HYALs, HYAL1 and HYAL2 expressed in chondrocytes can degrade CS as endogenous hydrolase, but they can digest CS more slowly than HA^33,34^. Another possible candidate mechanism of CS degradation is dependent on ROS and NO which are elevated in OA tissue^35,36^. ROS has been reported to degrade a variety of GAG such as CS, HA and dermatan sulfate^35^. NO also can degrade CS among which C6S is more susceptible than C4S^36^. Even though ROS and NO have limited cleavage capacity for CS, the partial fragmentation can expose the sulfated residue and change the affinity as ligand to TLRs, resulting in catabolic phenotype of chondrocytes such as hypertrophy-like change and proteases production.

We observed that the treatment of NAC, an antioxidant, unexpectedly increased the expression of MMP-13 and NO production. Paradoxical increase of the oxidative stress markers such as Fth1 and Hmox1 in RNA level suggests that NAC failed to function as an antioxidant as it originally intended and rather increased oxidative stress (Fig. 3). This can be attributed to the iNOS increase by NAC and subsequent increase of NO production. NAC has been known to have two opposing effect on NO generation depending on whether it is a physiologic or pathologic environment^37–39^. If LPS is given in excess in sepsis model, NAC reduced NO production along with the reduction of iNOS^37,38^. However, NAC increased NO generation in renal vascular smooth muscle cells in physiologic condition^39^. Our result showed that the treatment of NAC increases the expression of iNOS in chondrocytes as well as NO concentration in supernatant (Fig. 3B,C). NO can inhibit the activity of several enzymes of the mitochondrial respiratory chain including complex I, II–III, and IV^40^. The inhibition of mitochondrial respiration by NO may increase the electron leakage, and cause the formation of endogenous ROS^41^. It is possible that NAC-mediated NO production can paradoxically increase ROS generation.

When NO generation was suppressed with iNOS inhibitor, the activation of p38, JNK, ERK1/2 MAP kinases has been delayed and that of IkB was suppressed (Fig. 4A). NO has been reported to regulate the activation of MAP kinases and NFkB signaling which are an important upstream regulator of MMP-13 and ADAMTS5 production^42–45^. NO is known to activate p38, JNK, ERK1/2 MAP kinases via cGMP-mediated activation of protein kinase G^42,43^. Recent study also showed that NO activates ERK signaling through down-regulation of MAP kinase phosphatase^46^. With regard to NFkB, the peroxynitrite produced by NO and ROS can increase the cytokine-mediated nuclear translocation of NFkB in chondrocytes^45^. In turn, both p38 MAP kinase and NFkB can be involved in the transcription of iNOS^47,48^. These evidences suggest that NO interacts with both MAP kinases and NFkB signaling and this can accentuates the catabolic phenotypes of chondrocytes.

In conclusion, the degradation of CS-based ECM alone is enough to induce the hypertrophy-like morphological changes as well as the production of MMP-13 and ADAMTS5 in chondrocytes. Breakdown products of chondroitin-sulfate act as DAMPs via TLR2 and TLR4 enabling the activation of MAP kinases, NFkB and STAT3 which form a positive feedback loop with NO signaling (Fig. 4B). Our data provide an insight to understand the role of GAG degradation during cartilage degradation.

## Materials and Methods

### Primary chondrocyte culture

Primary chondrocytes were isolated from long bones of C57BL/6 E15.5 mice as previously described^49^. Bones (humerus, radius, femur and tibia) were isolated and equilibrated in α-MEM-based organ culture media supplemented with 0.2% BSA, 0.25 mM ascorbic acid, 1 mM β-glycerophosphate, 0.25% L-glutamin and 0.25% penicillin/streptomycin at 37 °C with 5% CO2 for overnight. They were incubated in trypsin-EDTA with gentle rocking for 15 minutes at 37 °C and digested with 3 mg/ml of collagenase P (Worthington, Lakewood, NJ) in DMEM containing 10% FBS for 2 hours. The fragmented long bones were filtered through a 40-μm nylon mesh (BD Bioscience, San Jose, CA), and cells were collected by centrifugation. Cells were cultured in 2:3 DMEM: F12 medium with 10% fetal bovine serum (FBS), and 0.25% L-glutamine. Primary chondrocytes at passage 0 were used for experiments to minimize dedifferentiation.

All animal protocols were approved by the Institutional Animal Care and Use Committee of the Daegu Fatima Hospital (approved protocol number F-17–2). Mice were purchased from Koatech Animal Inc. (Pyeongtaek, Korea) and maintained in standard laboratory conditions at the Daegu Fatima Hospital and conformed to the Guide for the Care and Use of Laboratory Animals published by the US National Institutes of Health (NIH publication, 8th Edition, 2011).

### Primary chondrocyte-laden 3D hydrogel culture and treatment of chondroitinase ABC

Chondroitin sulfate-methacrylate (CS-MA) polymer was synthesized as described previously^50^. Briefly, 2-morpholinoethanesulfonic acid (MES, Sigma-Aldrich, St. Louis, MO) and sodium chloride were dissolved in distilled water completely by stirring. Chondroitin sulfate sodium (Sigma-Aldrich), N-hydroxysuccinimide (NHS, Sigma-Aldrich) and 1-ethyl-3-(3-dimethylaminopropyl)-carbodiimide hydrochloride (EDC, Sigma-Aldrich) were dissolved in upper solution and stirred for 5 minutes, and added 2-aminoethyl methacrylate (AEMA, Sigma-Aldrich). The reaction mixture were maintained for 24 hours in room temperature protected from the light, and dialyzed against distilled water for 4 days followed filtration by 0.22 μm of syringe filter. The result CS-MA was dried in a vacuum oven at room temperature for overnight and stored in −20 °C. For chondrocyte encapsulation, the poly (ethylene glycol diacrylate) (PEGDA, Laysan Bio), CS-MA polymer and photo initiator (2-Hydroxy-4′-(2-Hydroxoyethoxy)-2-methylpropane (Sigma-Aldrich)) were dissolved in PBS completely by vortexing, and added primary chondrocytes at a density of 1.5 × 10^7^ cells/ml. After mix the hydrogel solution containing primary chondrocytes 30 times thoroughly, pipette 100 μl of the cell-hydrogel suspension into PCR film-sealed custom-made gel mold and polymerized by exposing to UV (365 nm wavelength) at 3 mW/cm^2^ for 10 minutes using long wave ultraviolet lamp (UVP, LLC, California, USA). Remove the PCR film from the gel mold and push out the gel into 100 mm dish containing PBS by cylindrical rods. Cell-laden hydrogels were cultured in 24-well plate with 1.5 ml of primary chondrocyte growth media for 3 weeks. Chondroitinase ABC (ChABC; 0.1 unit/ml, Sigma-Aldrich) was treated for the last 1 or 2 week in the presence of various inhibitors including 30 μg/ml of OxPAPC for TLR2/4, and 10 μg/ml LPS-RS for TLR4-MD2 from Invitrogen, 10 μM SB203580 for p38 MAPK from Sigma-Aldrich, 20 nM PD98059 for ERK1/2 MAPK from Cell Signaling Technology, and 20 μM SP600125 for JNK MAP kinase, 10 μM JSH-23 for NF-kB, 50 μM 1400 w for NOS, 3 mM N-acetyl-cysteine (NAC) for ROS and 5 μM Stattic for Stat3 from Sigma-Aldrich. Media were changed at every 3 days.

### Real-time qPCR analysis

Total RNA was isolated from cultured primary chondrocytes-laden 3D hydrogel using Easy Blue reagent (iNtRON biotechnology, Korea). The cDNA was synthesized from 2 μg of total RNA using reverse transcription kit (ELPIS Biotech, Korea) with random hexameter and oligo d(T) primer, and real-time qPCR was performed using the SYBR green master mix on the ViiA7 machine (Applied Biosystems, Waltham, MA). The expression of target genes was normalized to Gapdh. The sequences of primers used for RT-qPCR are listed in Supplementary Table 1.

### Frozen section and H&E staining

Cultured chondrocyte-laden hydrogels were washed with PBS and fixed with 4% paraformaldehyde (Merck, Darmstadt, Germany). Fixed hydrogels were covered entirely with OCT compound (Thermo Fisher Scientific, Waltham, MA) on to a tissue base mold and frozen in cryomold with 2-methyl-butane (Thermo Fisher Scientific) cooled by dry ice. 5-μm sections were made by Cryostat (Micron HM350, Thermo Fisher Scientific). The sections were air-dried overnight at room temperature and then stained with hematoxin and eosin.

### Immunofluorescence staining

Microdisected frozen sections of cell-laden hydrogels were performed immunofluorescence staining to detect the expression of Mmp13, Adamts5 and 8-oxo-dG. Briefly, dried sections in 37 °C oven for 1 hour were fixed with cold acetone for 20 minutes and washed with PBS twice. After blocking with 1% BSA for 1 hour, sections were incubated with primary antibodies against Mmp13 (ab39012), Adamts5 (ab41037, Abcam, Cambridge, UK) for detection of chondrocyte differentiation status, or 8-oxo-deoxyguanosine (4354-MC-050, Trevigen, Gaithersburg, MD) for detection of DNA oxidation to measurement of oxidative stress at 4 °C for overnight. The sections were washed with PBS, incubated with an Alexa Fluor 488- or 594-conjugated secondary antibodies (Thermo Fisher Scientific) for 1 hour and counter-stained with DAPI. Sections were mounted with anti-fade mounting solution (Invitrogen, Carlsbad, CA), and imaged and quantified under Nikon ECLIPSE Ni fluorescence microscope (Nikon, Japan).

### Nitric oxide assay

The nitric oxide colorimetric assay was performed as described previously^51^. Briefly, the primary chondrocytes-laden hydrogels were cultured for 3 weeks and the quantity of nitrite, a stable metabolite of NO, in culture medium was measured as an indicator of NO production. The 100 μl of cultured media was mixed with same amount of Griess reagent (Cell Signaling Technology, Danvers, MA) in 96 well plate and incubated at room temperature for 10 minutes, and measured the absorbance at 540 nm in a microplate reader (BioTek, Winooski, VT). Fresh culture medium was used as a blank in every experiment. The quantity of nitrite was determined from a sodium nitrite standard curve.

### TLR4 signaling activation with lipopolysaccharides (LPS) and Western blot analysis

Primary chondrocytes were treated with 1 μg/ml of LPS (Sigma-Aldrich) for indicated time points (0, 5, 15, 30, 60 and 120 min) in the presence or absence of 50 μM of 1400 w, and then lysed with RIPA buffer supplemented with protease and phosphatase inhibitor (Roche Diagnostics, Indianapolis, IN). Equal amount of cell lysates from each samples were separated by 10% SDS-PAGE and transferred to PVDF membranes (Amersham Biosciences, Buckinghamshire, UK). The Membranes were blocked with 5% skim milk at RT for 1 hour, followed by incubation with the primary antibodies listed below for 1 hour with shaking. After washing the membrane with TBS-T, the membranes were incubated with an HRP-conjugated secondary antibody (Santa Cruz Biotechnology, CA, USA) for 1 hour and washed again. The signal on the membranes was visualized using enhanced chemiluminescence solution (Amersham Biosciences) and detected in FluorChem FC2 (Alpha Inotech). The primary antibodies against p-p38 (Thr180/Tyr182), p-Jnk (Thr183/Tyr185), p-Erk1/2 (Thr202/Tyr204), p-Ikb (Ser32/36), p-Stat3 (Tyr705) were purchased from Cell Signaling Technology and β-Actin was from Sigma-Aldrich.

### Statistical analyses

The Mann-Whitney U tests or Kruskal–Wallis one-way analysis of variance (ANOVA) tests were used to determine differences between means. All analyses were conducted using SPSS version 14.0 software (SPSS, Chicago, IL). Results are presented as means ± S.Es and statistical significance was accepted for p values of <0.05.

### Ethics approval and consent to participate

All animal protocols were approved by the Institutional Animal Care and Use Committee of the Daegu Fatima Hospital (approved protocol number F-16–04).

## Supplementary information

Supplementary dataset

## Data Availability

Please contact author for data requests.
