# Supplementary material for: Degrading products of chondroitin sulfate can induce hypertrophy-like changes and MMP-13/ADAMTS5 production in chondrocytes
Source: Sci Rep. 2019 Nov 1;9:15846. doi: 10.1038/s41598-019-52358-4 (PMC6825126; doi:10.1038/s41598-019-52358-4)
Supplement: Supplementary file 1 — Supplementary dataset [file 41598_2019_52358_MOESM1_ESM.docx]

**Degrading products of chondroitin sulfate can induce hypertrophy-like changes and MMP-13/ADAMTS5 production in chondrocytes**

Youn-Kwan Jung, PhD^1^, Hye-Ri Park, MS^2^, Hyun-Jung Cho, MS ^2^, Ji-Ae Jang, MS^2^, Eun-Ju Lee, MS ^2^, Min-Su Han, PhD ^2^, Gun-Woo Kim, MD, PhD,^2,3^, and Seungwoo Han, MD, PhD ^2,4^

^1^Biomedical Research Institute, Gyeongsang National University Hospital, Jinju, Gyeongsangnam-do, Republic of Korea

^2^Laboratory for arthritis and bone biology, Fatima Research Institute, ^3^Department of Internal medicine, Daegu Fatima Hospital, Daegu, Republic of Korea

^4^Department of Internal medicine, Kyungpook National University Hospital, Daegu, Republic of Korea

**Supplementary figure legends**

Supplementary figure 1. Chondroitin-sulfate-based 3D hydrogel culture conditions are suitable for the hypertrophic differentiation of primary chondrocytes. (A) A mixture of chondroitin sulfate and poly(ethylene glycol) with 1.5x10^7^/ml of primary chondrocytes was allowed to form gels through exposure to 365 nm wavelength UV light for the indicated times. (B) Primary chondrocytes can differentiate into a hypertrophy-like phenotype through in vitro 3D hydrogel culture for a 7-week period. H&E and Safranin-O staining of frozen-sectioned 3D hydrogel harvested at the indicated time points. Safranin-O staining showed that the glycosaminoglycan content of ECM is dyed up to 3 weeks but not after 5 weeks of culture. Then, hypertrophy-like morphologic changes in chondrocytes were observed. (C) Real-time qRT-PCR analysis of chondrocyte-specific marker genes for chondrogenic differentiation, such as Sox9, Col2 and Agc; the pre-hypertrophy gene Ihh; the early hypertrophy genes Col10 and Runx2; the late hypertrophy gene MMP-13; and the osteoblast marker Col1a1.


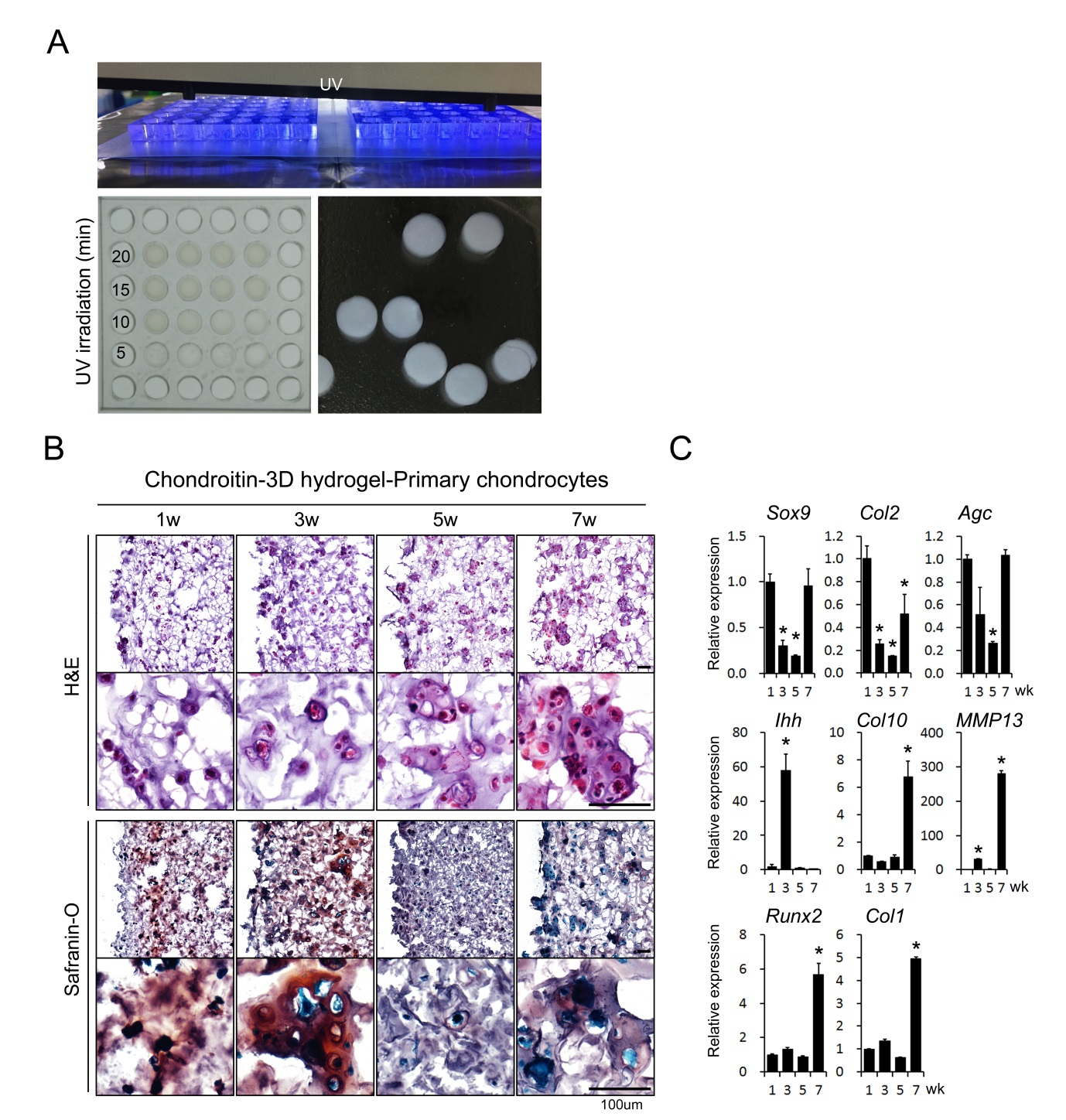


Supplementary figure 2. In a chondroitin sulfate-free poly(ethylene glycol) (PEG) hydrogel, chondroitinase does not induce morphological changes and transcription of catalytic protease and oxidative stress-related genes in primary chondrocytes. Primary chondrocytes (1.5x10^7^/ml) were cultured in PEG hydrogel which did not contain chondroitin sulfate for 1 week and then treated with chondroitinase ABC for 1 week. (A) H&E staining of frozen-sectioned 3D hydrogel. (B) Real-time qRT-PCR analysis of proteases such as MMP-13 and ADAMTS5, and oxidative stress-related genes, FTH1, Hmox1, and Txn, and iNOS.


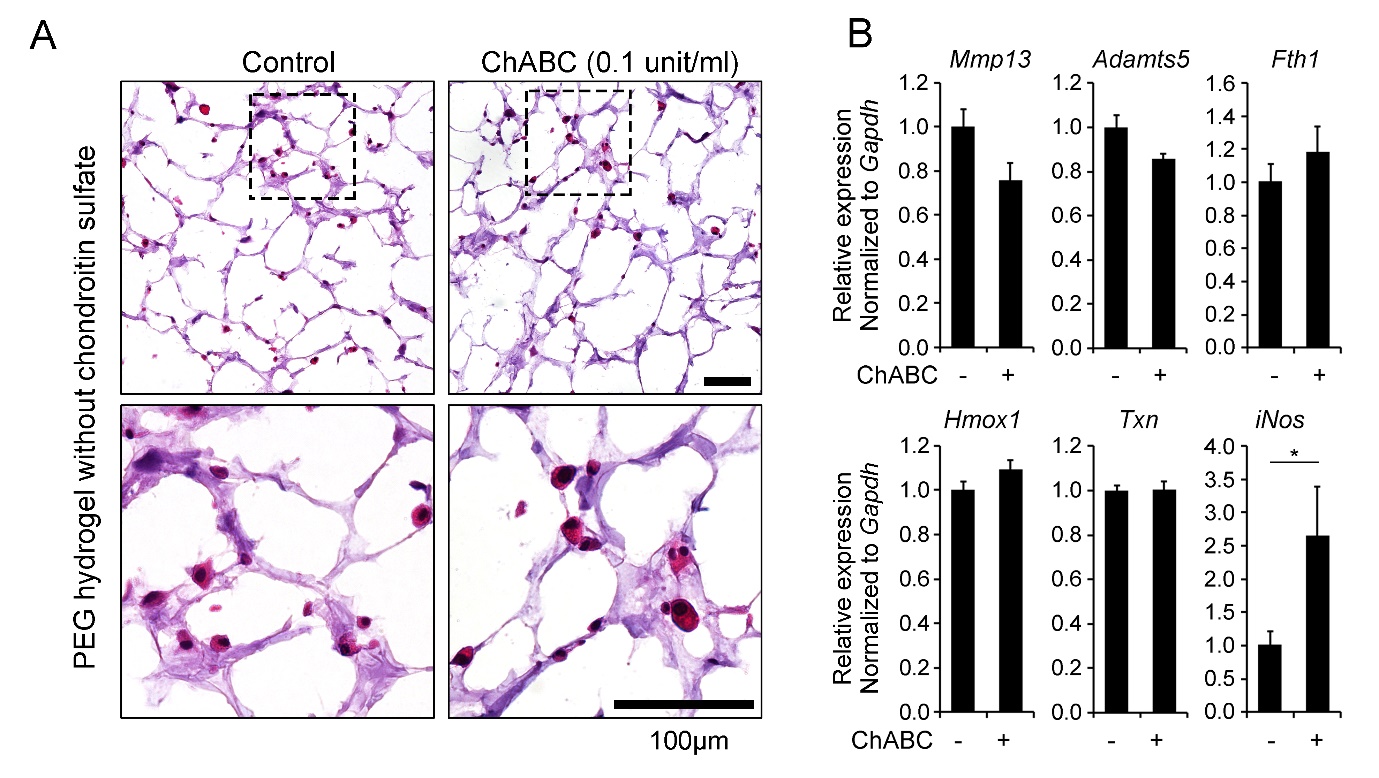


Supplementary figure 3. The breakdown of chondroitin sulfate and the expression of metabolism-associated genes. Primary chondrocytes were treated with chondroitinase ABC for 1 week and 2 weeks, and cells were harvested for total RNA isolation and subjected to real-time qRT-CRP to determine the changes in RNA expression of metabolism-associated genes.


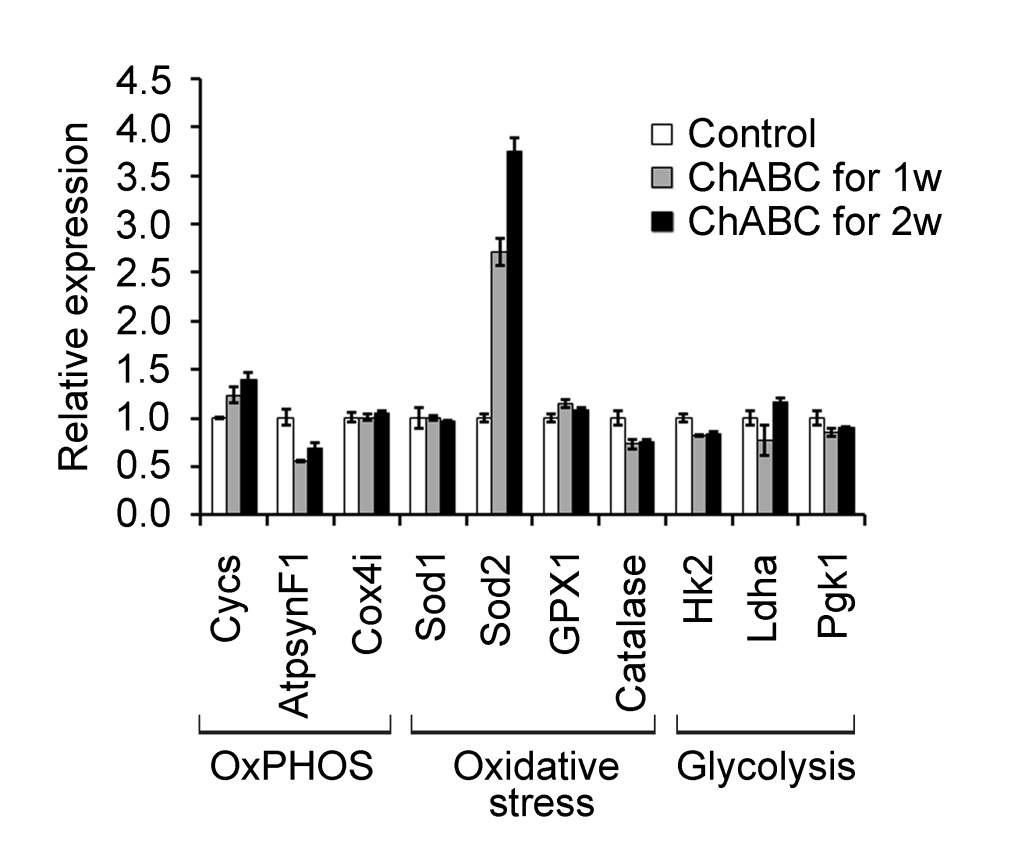


Supplementary figure 4. Full sized result of Western blot in figure 4A.

**
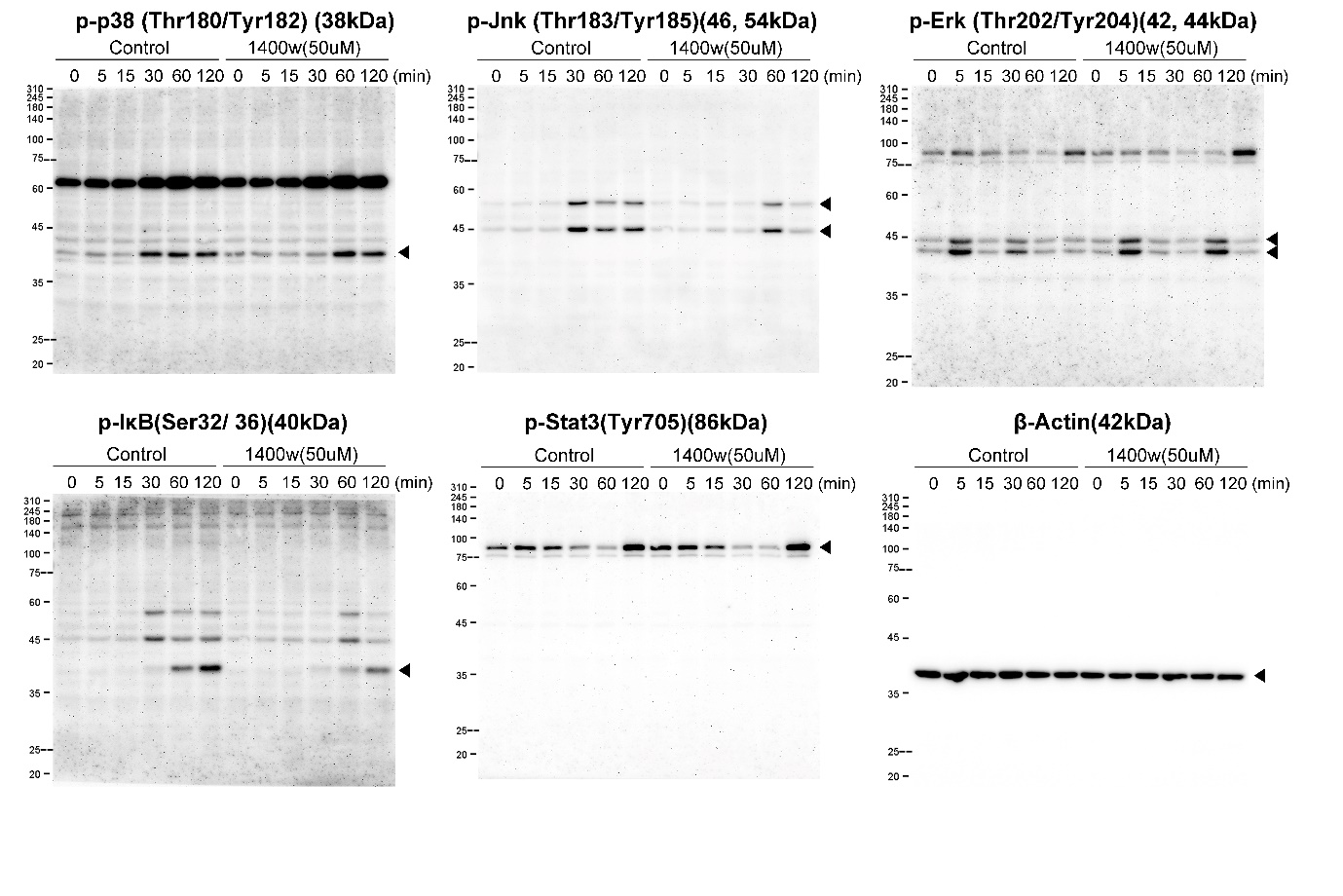
**

Supplementary Table 1. Primer sequences for the real-time quantitative PCR analysis

| Gene | GenBank Acc. No. | Sequences | Amplicon (bp) |
| --- | --- | --- | --- |
| *Sox9* | NM_011448 | Forward: 5'-CAGCCCCTTCAACCTTCCTC-3'  Reverse: 5'-TGATGGTCAGCG- TAGTCGTATT-3' | 94 |
| *Col2* | NM_031163 | Forward: 5'-CGGTCCTACGGTGTCAGG-3'  Reverse: 5'-TTATACCTCTGCCCATTCTGC-3' | 70 |
| *Agc* | NM_007424 | Forward: 5'-CCAGCCTACACCCCAGTG-3'  Reverse: 5'-GAGGGTGGGAAGCCATGT-3' | 66 |
| *Ihh* | NM_010544 | Forward: 5'-TGCATTGCTCTGTCAAGTCTG-3'  Reverse: 5'-GCTCCCCGTTCTCTAGGC-3' | 93 |
| *Col10* | NM_009925 | Forward: 5'-GCATCTCCCAGCACCAGA-3'  Reverse: 5'-CCATGAACCAGGGTCAAGAA-3' | 85 |
| *Mmp13* | NM_008607 | Forward: 5'-GCCAGAACTTCCCAACCAT-3'  Reverse: 5'-TCAGAGCCCAGAATTTTCTCC-3' | 92 |
| *Runx2* | NM_001146038 | Forward: 5'-GCCCAGGCGTATTTCAGA-3'  Reverse: 5'-TGCCTGGCTCTTCTTACTGAG-3' | 82 |
| *Col1a1* | NM_007742 | Forward: 5'-CTTCACCTACAGCACCCTTGTG-3'  Reverse: 5'-TTGGTGGTTTTGTATTCGATGACT-3' | 80 |
| *Atf4* | NM_009716 | Forward: 5'-TCGATGCTCTGTTTCGAATGG-3'  Reverse: 5'-AAGATCACATGTGTCATCCAACGT-3' | 80 |
| *Gadd45b* | NM_008655 | Forward: 5'-GAGGATGATATCGCTCTGCAGAT-3'  Reverse: 5'-ACCCGGACGATGTCAATGTC-3' | 80 |
| *Axin2* | NM_015732 | Forward: 5'-GACGCACTGACCGACGATT-3'  Reverse: 5'-TTCTTACTCCCCATGCGGTAA-3' | 80 |
| *Wisp1* | NM_018865 | Forward: 5'-GACCTGACCGAGTTGCCTAATG-3'  Reverse: 5'-GCGAAGTCTTCCCCATCGT-3' | 80 |
| *Wnt5a* | NM_009524 | Forward: 5'-CATTGGAGAAGGTGCGAAGAC-3'  Reverse: 5'-CCACTGTGCTGCAGTTCCAT-3' | 80 |
| *Csf1* | NM_007778 | Forward: 5'-TCCTCATGAGCAGGAGTATTGC-3'  Reverse: 5'-CAGGACCTTCAGGTGTCCATTC-3' | 80 |
| *Stat1* | NM_001205313 | Forward: 5'-TGAGATGTCCCGGATAGTGG-3'  Reverse: 5'-CGCCAGAGAGAAATTCGTGT-3' | 80 |
| *Irf1* | NM_008390 | Forward: 5'-GGAAGGGAAGATAGCCGAAGA-3'  Reverse: 5'-ATCCCTTGCCATCGATGTGT-3' | 80 |
| *Socs3* | NM_007707 | Forward: 5'-CCGCTTCGACTGTGTACTCAAG-3'  Reverse: 5'-CCGTGGGTGGCAAAGAAA-3' | 80 |
| *Gata3a* | NM_008091 | Forward: 5'-CGGAAGAGGTGGACGTACTTTT-3'  Reverse: 5'-CGTAGCCCTGACGGAGTTTC-3' | 80 |
| *Bax* | NM_007527 | Forward: 5'-GTGAGCGGCTGCTTGTCT-3'  Reverse: 5'-GGTCCCGAAGTAGGAGAGGA-3' | 68 |
| *Ccnd1* | NM_007631 | Forward: 5'-GCGTACCCTGACACCAATCTC-3'  Reverse: 5'-CTCCTCTTCGCACTTCTGCTC-3' | 80 |
| *Pcna* | NM_011045 | Forward: 5'-TTTGAGGCACGCCTGATCC-3'  Reverse: 5'-GGAGACGTGAGACGAGTCCAT-3' | 80 |
| *Hes1* | NM_008235 | Forward: 5'-CCAGCCAGTGTCAACACGA-3'  Reverse: 5'-AATGCCGGGAGCTATCTTTCT-3' | 80 |
| *Hey1* | NM_010423 | Forward: 5'-GCGCGGACGAGAATGGAAA-3'  Reverse: 5'-TCAGGTGATCCACAGTCATCTG-3' | 80 |
| *Ptch1* | NM_008957 | Forward: 5'-GGAAGGGGCAAAGCTACAGT-3'  Reverse: 5'-TCCACCGTAAAGGAGGCTTA-3' | 62 |
| *Gli1* | NM_010296 | Forward: 5'-CTGACTGTGCCCGAGAGTG-3'  Reverse: 5'-CGCTGCTGCAAGAGGACT-3' | 64 |
| *Hhip* | NM_020259 | Forward: 5'-CCCAGACTCACAATGGAAAACTCT-3'  Reverse: 5'-GGTGTAGTAGCCGTTTCGACAGA-3' | 80 |
| *Fth1* | NM_010239 | Forward: 5'-TGACCACGTGACCAACTTACG-3'  Reverse: 5'-CCAGGGTGTGCTTGTCAAAGA-3' | 80 |
| *Hmox1* | NM_010442 | Forward: 5'-TGACCACGTGACCAACTTACG-3'  Reverse: 5'-CCAGGGTGTGCTTGTCAAAGA-3' | 80 |
| *Txn* | NM_011660 | Forward: 5'-ATCAAGCCCTTCTTCCATTCC-3'  Reverse: 5'-TCCTGGCAGTCATCCACATC-3' | 80 |
| *Arnt* | NM_001037737 | Forward: 5'-CCTGGCTCGAAAACCAGACA-3'  Reverse: 5'-TGTTGCCAGTTCCCCTCAAG-3' | 80 |
| *Serpine1* | NM_008871 | Forward: 5'-ATGACTGGGTGGAAAGGCATAC-3'  Reverse: 5'-CAGGCGTGTCAGCTCGTCTA-3' | 80 |
| *Vegfa* | NM_001025250 | Forward: 5'-GCAGCTTGAGTTAAACGAACG-3'  Reverse: 5'-GGTTCCCGAAACCCTGAG-3' | 94 |
| *Cycs* | NM_007808 | Forward: 5'-GGTGATGTTGAAAAAGGCAAGAA-3'  Reverse: 5'-TTATGCTTGCCTCCCTTTTCC-3' | 80 |
| *AtpsynF1* | NM_007505 | Forward: 5'-CCTGGAATTATCCCCCGAAT-3'  Reverse: 5'-CCAATGGGCACCAGGCTAT-3' | 80 |
| *Cox4i* | NM_009941 | Forward: 5'-TCACTGCGCTCGTTCTGAT-3'  Reverse: 5'-CGATCGAAAGTATGAGGGATG-3' | 80 |
| *Pepck* | NM_011044 | Forward: 5'-CTGGCCAAGATTGGTATTGAACT-3'  Reverse: 5'-GATATGCCCATCCGAGTCATG-3' | 80 |
| *G6pase* | NM_008061 | Forward: 5'-CATGTACCGGAAGAGCTGCAA-3'  Reverse: 5'-GGACCAAGGAAGCCACAATG-3' | 80 |
| *Sod1* | NM_011434 | Forward: 5'-GCGGTGAACCAGTTGTGTTG-3'  Reverse: 5'-CCCATACTGATGGACGTGGAA-3' | 80 |
| *Sod2* | NM_013671 | Forward: 5'-CCACACATTAACGCGCAGAT-3'  Reverse: 5'-TCGGTGGCGTTGAGATTGT-3' | 80 |
| *Gpx1* | NM_008160 | Forward: 5'-AAGTGCGAAGTGAATGGTGAGA-3'  Reverse: 5'-GGGTCGTCACTGGGTGTTG-3' | 80 |
| *Catalase* | NM_009804 | Forward: 5'-GGCCTCGCAGAGACCTGAT-3'  Reverse: 5'-ACCCCGCGGTCATGATATTA-3' | 80 |
| *Hk2* | NM_013820 | Forward: 5'-CACGGAGCTCAACCAAAACC-3'  Reverse: 5'-CCGGAACCGCCTAGAAATCT-3' | 100 |
| *Ldha* | NM_010699 | Forward: 5'-CTCAAGGACCAGCTGATTGTGA-3'  Reverse: 5'-GCACCAACCCCAACAACTGT-3' | 80 |
| *Pgk1* | NM_008828 | Forward: 5'-TGGACAAGCTGGACGTGAAG-3'  Reverse: 5'-GCCTTGATCCTTTGGTTGTTTG-3' | 100 |
| *iNos* | NM_010927 | Forward: 5'-GCAAGCATGACTTCCGAGTGT-3'  Reverse: 5'-GCCCAAGGTAGAGCCATCTG-3' | 100 |
| *Gapdh* | NM_008084 | Forward: 5'-TGTCCGTCGTGGATCTGAC-3'  Reverse: 5'-CCTGCTTCACCACCTTCTTG-3' | 75 |
